# Alterations in Immune-Related Genes as Potential Marker of Prognosis in Breast Cancer

**DOI:** 10.3389/fonc.2020.00333

**Published:** 2020-03-12

**Authors:** Bei Li, Rongxin Geng, Qi Wu, Qian Yang, Si Sun, Shan Zhu, Zhiliang Xu, Shengrong Sun

**Affiliations:** ^1^Department of Breast and Thyroid Surgery, Renmin Hospital of Wuhan University, Wuhan, China; ^2^Department of Neurosurgery, Renmin Hospital of Wuhan University, Wuhan, China; ^3^Brain Tumor Clinical Center of Wuhan, Wuhan, China; ^4^Faculty of Medicine, University of Paris Sud-Saclay, Le Kremlin-Bicêtre, France; ^5^Department of Clinical Laboratory, Renmin Hospital of Wuhan University, Wuhan, China

**Keywords:** breast cancer, immune score, stromal score, overall survival, aging

## Abstract

The tumor microenvironment (TME) is a heterogeneous system that contributes to breast cancer progression. The Cancer Genome Atlas (TCGA) database provides global gene expression profiling data for further analysis of various malignancies, including breast cancer. Based on the ESTIMATE algorithm, immune and stromal scores were calculated according to immune or stromal components in the TME. We divided breast cancer cases into high- and low-score groups and identified differentially expressed genes (DEGs) that were significantly associated with overall survival. We performed enrichment analysis and constructed a protein-protein interaction network and found that the DEGs were mainly involved in primary immunodeficiency, T cell receptor signaling pathway and cytokine-cytokine receptor reaction. Furthermore, we explored the effect of aging on immune and stromal scores, which was validated by lower immune/stromal scores, lower infiltration of T cells and lower expression of immune checkpoints in the elder group. In conclusion, certain differentially expressed immune-related genes contribute to longer overall survival, and aging influences the immune microenvironment and immunotherapy efficacy by changing the tumor-infiltrating lymphocyte (TIL) abundance and checkpoint expression in breast cancer.

## Introduction

Breast cancer is the most common malignant tumor in women, with an incidence that increases yearly, and it has the second highest mortality rate among malignant tumors in women (1). Breast cancer is a heterogeneous disease with variable molecular characteristics and discrepant components in the tumor microenvironment (TME). As an extremely heterogeneous system, the TME is composed of infiltrating lymphocytes, macrophages, fibroblasts, extracellular matrix, adipocytes, and other stromal components (2, 3). In addition, the TME has been well recognized as an essential factor in cancer development, growth, and progression (2). In the microenvironment, in addition to tumor cells, immune cells and stromal cells are the major cell types that reprogram the inflammatory and metabolic profiles of cancer cells (3, 4). However, the transcriptome profile of cancer involves parameters derived from both tumor cells and immune cells and stromal cells in the tumor microenvironment. Indeed, previous studies have demonstrated immune or stromal “signatures” in breast cancer, which are possibly relevant to clinical features (5, 6). Thus, it is speculated that the gene expression profile of microenvironment components may impact the specific subtypes of breast cancer. Importantly, whether and to what extent microenvironment effects result in assigning breast cancer to a specific phenotype have not been assessed.

Age is considered a pivotal risk factor for breast cancer, which has become a huge health burden in the senescent population (7). Further, aging can remarkably contribute to breast cancer progression and is associated with a worse disease course (8). Aging and cancer are two highly related biological phenomena, however, the causative relationship between them remains unclear and requires more study. Although many studies have been focused on investigating the mechanism why aging promotes oncogenesis, less attention has been paid to explore the special characteristics of elderly patients with cancer. It is still unknown whether aging can induce unique molecular features and whether aging-associated features should shape treatment strategies in elderly cancer patients.

Based on the global gene expression data in The Cancer Genome Atlas (TCGA) database, algorithms have been developed to calculate tumor purity, such as ESTIMATE (Estimation of Stromal and Immune cells in Malignant Tumor tissues using Expression data) (9). ESTIMATE is an algorithm that calculates immune and stromal scores of cancer to predict the infiltration of immune and stromal cells by analyzing specific gene expression profiles of immune and stromal cells (9). Therefore, we designed an analytical procedure to perform a pan-cancer transcriptomic analysis and investigated senescence-related altered immune cells and immune checkpoints in the tumor microenvironment.

## Materials and Methods

### Database

The gene expression data of breast cancer patients were downloaded from TCGA data portal (https://tcga-data.nci.nih.gov/tcga/). Clinical information, including age, hormone receptor status and survival, were also obtained from TCGA data portal. We calculated immune scores and stromal scores by applying the ESTIMATE algorithm to gene expression data (9).

### Recognition of Differentially Expressed Genes (DEGs)

Differentially expressed genes between the high immune/stromal score group and low immune/stromal score group were detected using the package limma (10). To reduce the false positive rate, we used the Benjamini and Hochberg false discovery rate to adjust *p*-values. An adjusted *p* < 0.05 and |logFC| > 1 were defined as the cutoff criteria. Additionally, we used ImageGP (http://www.ehbio.com/ImageGP/index.php/Home/Index/index.html) to construct a visual volcano plot to display the results of the dataset analysis and Morpheus online analysis software (https://software.broadinstitute.org/morpheus/) to construct a heat map and perform hierarchical cluster analysis.

### Acquisition of the Common DEGs

The common DEGs, which stemmed from the immune scores and stromal scores, were acquired using a Venn analysis. Subsequently, we constructed a visual Venn diagram to show the results of the intersection using Bioinformatics & Evolutionary Genomics online tools (http://bioinformatics.psb.ugent.be/webtools/Venn/).

### Enrichment Analysis of DEGs

Gene ontology (GO) analysis is an approach to annotate genes and gene products through analyzing high-throughput genome or transcriptome data (11). Kyoto Encyclopedia of Genes and Genomes (KEGG) is a database collection that can help in analysis of genomes, biological pathways, diseases, and drugs (12). The Database for Annotation, Visualization and Integrated Discovery (DAVID, https://david.ncifcrf.gov/) is an online bioinformatics tool providing a means for functional analysis of a number of genes and proteins (13). Significance levels were set at *p* < 0.05. We used DAVID to visualize the core biological process (BP), molecular function (MF), cellular component (CC), and pathways among the DEGs. Furthermore, we utilized ImageGP (http://www.ehbio.com/ImageGP/index.php/Home/Index/index.html) to construct a visual enrichment plot to display the outcomes.

### Construction of a PPI Network

Search Tool for the Retrieval of Interacting Genes (STRING) is an online tool that helps evaluate protein-protein interaction (PPI) networks (14). We used the STRING app in Cytoscape to analyze DEGs to identify potential relationships among the DEGs. We set the cutoff criteria as a confidence score ≥ 0.4 and maximum number of interactors = 0. Moreover, we utilized the Molecular Complex Detection (MCODE) app to screen modules in the PPI network in Cytoscape with the following criteria: degree cut-off = 2, node score cut-off = 0.2, k-core = 2, and max. depth = 100 (15). DAVID was used to perform pathway analysis of genes in these modules. To explore the potential information, we carried out GO and KEGG pathway analyses.

### Statistical Analysis

Overall survival (OS) curves of individual DEGs that were significantly associated with OS were downloaded from the website http://gepia.cancer-pku.cn/. Kaplan-Meier survival analyses of immune/stromal scores and age were performed using GraphPad Prism 8.0.1 software, *p*-values were calculated with a log-rank test, and *p* < 0.05 was set as the cut-off. All relevant statistical analyses were performed with SPSS 22.0 software (IBM Corporation, Armonk, NY, USA), and significance levels were set at *p* < 0.05.

## Results

### Immune Scores and Stromal Scores Are Significantly Associated With Breast Cancer Subtypes

**Figure 1:**
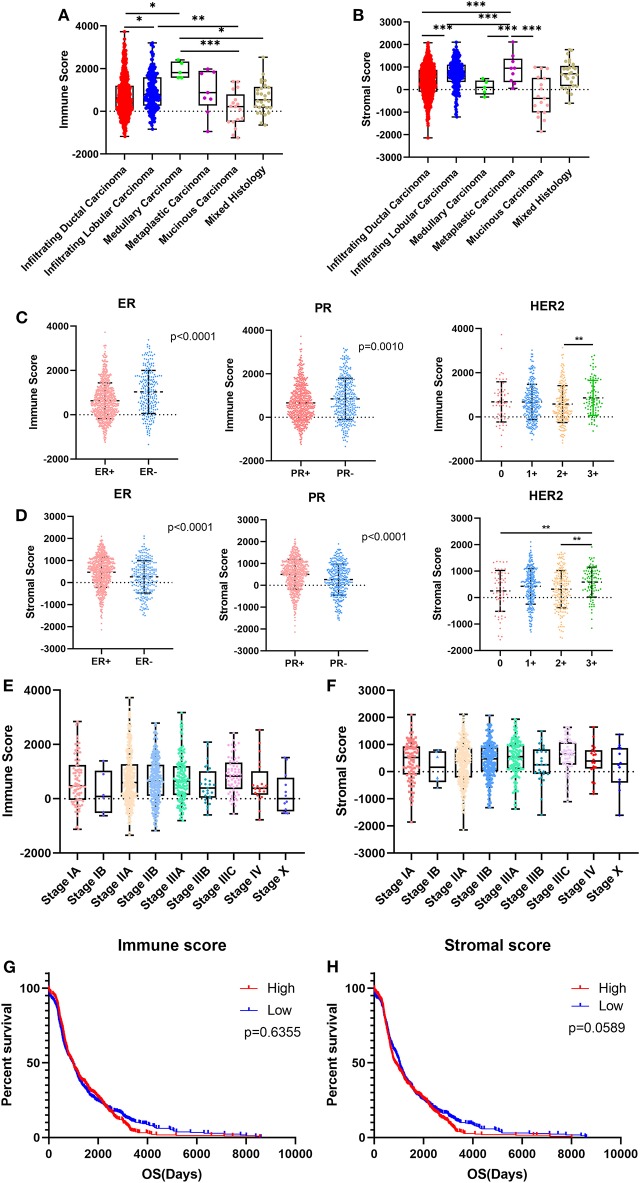
Immune scores and stromal scores are associated with breast cancer subtypes and patient overall survival. **(A,B)** Distribution of immune scores or stromal scores among breast cancer pathological subtypes. **(C,D)** Distribution of immune scores or stromal scores among breast cancer molecular subtypes. **(E,F)** Distribution of immune scores or stromal scores among breast cancer stages. **(G,H)** Patients were divided into high- and low-score groups according to their immune scores or stromal scores. Kaplan-Meier plots were generated to compare overall survival (days) in the high- and low-score groups. Differences were assessed with a log-rank test. *p < 0.05, **p < 0.01, and ***p < 0.001 for analysis of variance (ANOVA).

We downloaded gene expression data and clinical information of breast cancer patients from TCGA database. After exclusion of patients with incomplete clinical information and the normal control group, 1,089 breast cancer patients were included in our study. Based on the pathological diagnosis given in TCGA database, breast cancer was classified into five clear subtypes: infiltrating ductal carcinoma, medullary carcinoma, metaplastic carcinoma, infiltrating lobular carcinoma and mucinous carcinoma. The 1,089 cases included 779 (71.5%) infiltrating ductal carcinoma, 203 (18.6%) infiltrating lobular carcinoma, 5 (0.5%) medullary carcinoma, 9 (0.8%) metaplastic carcinoma, 17 (1.6%) mucinous carcinoma, 29 (2.7%) mixed histology, and 47 (4.3%) unknown subtypes. As analyzed using the ESTIMATE algorithm, immune scores ranged from −1342.120 to 3728.873, and stromal scores ranged from −2141.790 to 2112.231 (Figures 1A,B). As the most common types of breast cancer, the immune and stromal scores of infiltrating ductal carcinoma were lower than those of infiltrating lobular carcinoma (Figures 1A,B; *p* < 0.001, and *p* < 0.05, respectively). The mucinous carcinoma subtype had the lowest scores, whereas metaplastic carcinoma had the highest stromal score, and medullary carcinoma had the highest immune score, indicating that there was not much correlation between immune and stromal scores and the subtypes of breast cancer.

Given that this pathological classification of breast cancer is of limited clinical application, we focused on the molecular subtypes of breast cancer classified by hormone receptor status and human epidermal growth factor receptor-2 (HER2) status. For the immune score, cases with negative hormone receptor status and positive HER2 status had higher scores, whereas cases with positive hormone receptor status and positive HER2 status had higher stromal scores (Figures 1C,D).

We compared immune and stromal scores at different stages of breast cancer and found that there were no significant differences in either the immune or the stromal scores (Figures 1E,F).

We analyzed the potential association between overall survival (OS) and immune/stromal scores. According to the immune and stromal scores, we divided all 1,089 cases into two groups, a high-score group and low-score group. Kaplan-Meier survival curves showed that although the median OS of cases with low immune scores was shorter than that of cases with high immune scores the difference was not significant (Figure 1G; 965d vs. 975d, *p* = 0.6355). In the stromal score group, the median OS of cases with low stromal scores was longer than that of cases with high stromal scores, but again, the difference was not significant (Figure 1H; 1,025 d vs. 856 d, *p* = 0.0589). It seems that immune/stromal scores cannot significantly predict the OS of breast cancer patients.

### Comparison of Gene Expression Profiles With Immune Scores and Stromal Scores in Breast Cancer

**Figure 2:**
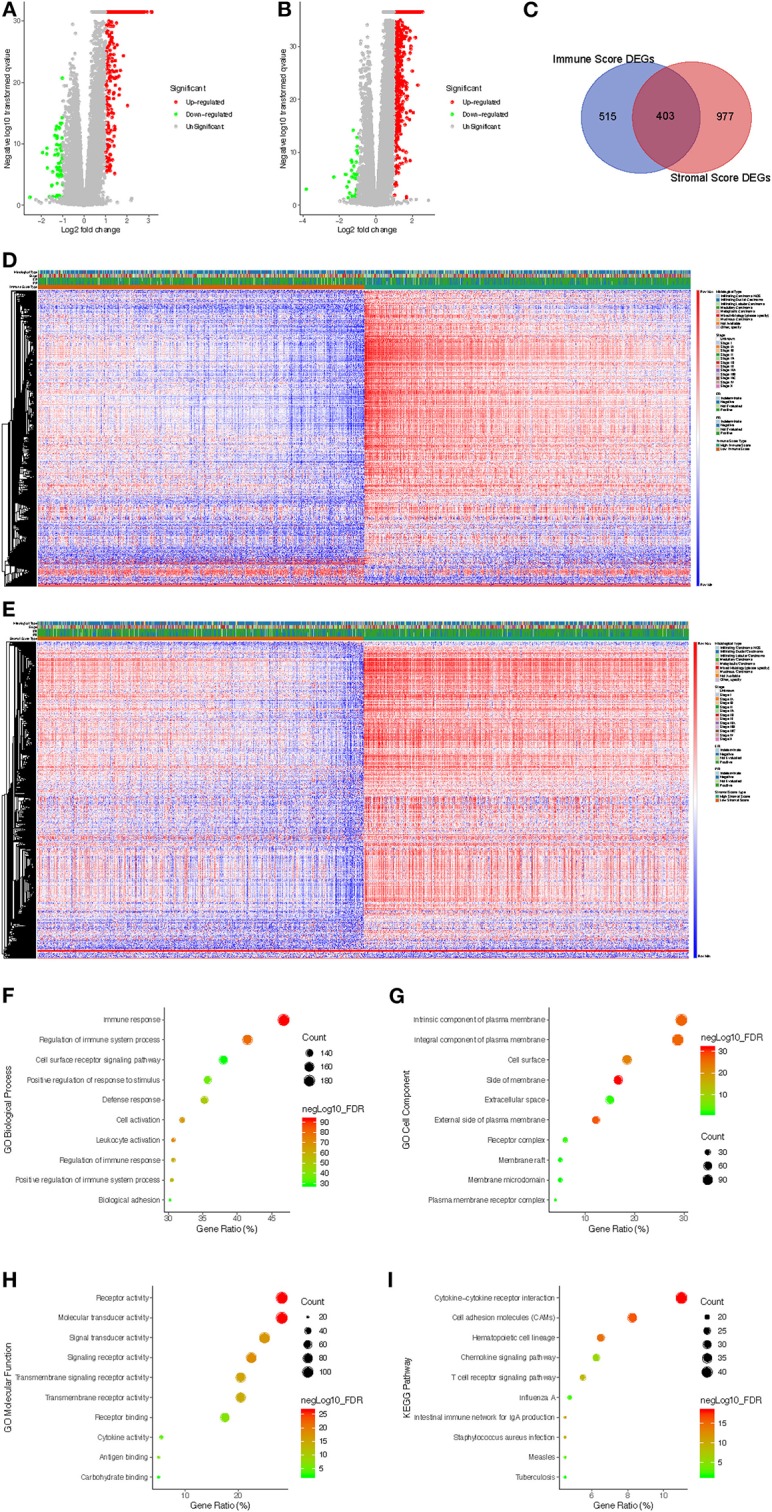
Comparison of gene expression profiles with immune scores and stromal scores in breast cancer. **(A)** The significantly upregulated and downregulated genes in the high immune score group compared with the low score group are shown in the volcano plot (fold change > 2.0, *p* < 0.05). **(B)** The significantly upregulated and downregulated genes in the high stromal score group compared with the low score group are shown in the volcano plots (fold change > 2.0, *p* < 0.05). **(C)** Venn diagrams showing the commonly upregulated genes in both the high immune score group and high stromal score group. **(D)** Immune score DEGs in breast cancer displayed in a heat map. **(E)** Stromal score DEGs in breast cancer displayed in a heat map. **(F–H)** GO analysis results showing that commonly upregulated DEGs were particularly enriched in BP **(F)**, CC **(G)**, and MF **(H)**. **(I)** The significantly enriched pathways of the commonly upregulated DEGs determined by KEGG analysis. GO, gene ontology; BP, biological process; CC, cell component; MF, molecular function; KEGG, Kyoto Encyclopedia of Genes and Genomes.

To investigate the association between gene expression profiles and immune/stromal scores, we analyzed gene expression data of all 1,089 breast cancer cases divided into high/low immune/stromal score groups. Based on immune scores, a volcano plot showed that 851 genes were upregulated and 67 genes were downregulated in the high-score group compared with the low-score group (Figure 2A; fold change > 2.0, *p* < 0.05). In the high stromal score group, 1,342 genes were upregulated, and 38 genes were downregulated (Figure 2B; fold change > 2.0, *p* < 0.05). In addition, a Venn diagram demonstrated that 397 genes were commonly upregulated while only 6 genes were commonly downregulated in the high-score groups (Figure 2C). Heatmaps also validated these differentially expressed genes (DEGs) (Figures 2D,E). To explore the function and pathway of these DEGs, we performed gene ontology (GO) function analysis using DAVID and KEGG pathway enrichment analysis. The GO results showed that DEGs were particularly enriched in the following biological processes (BPs): immune response, regulation of immune system process and cell surface receptor signaling pathway (Figure 2F). GO cell component (CC) analysis demonstrated that DEGs were enriched in integral component of plasma membrane, intrinsic component of plasma membrane and cell surface (Figure 2G). GO molecular function results showed that DEGs were enriched in molecular transducer activity, receptor activity and signal transducer activity (Figure 2H). The significantly enriched pathways shown by KEGG pathway analysis included cytokine-cytokine receptor reaction, hematopoietic cell lineage cell, adhesion molecules (CAMs) and chemokine signaling pathway (Figure 2I).

### Association Between DEGs and Overall Survival

**Figure 3:**
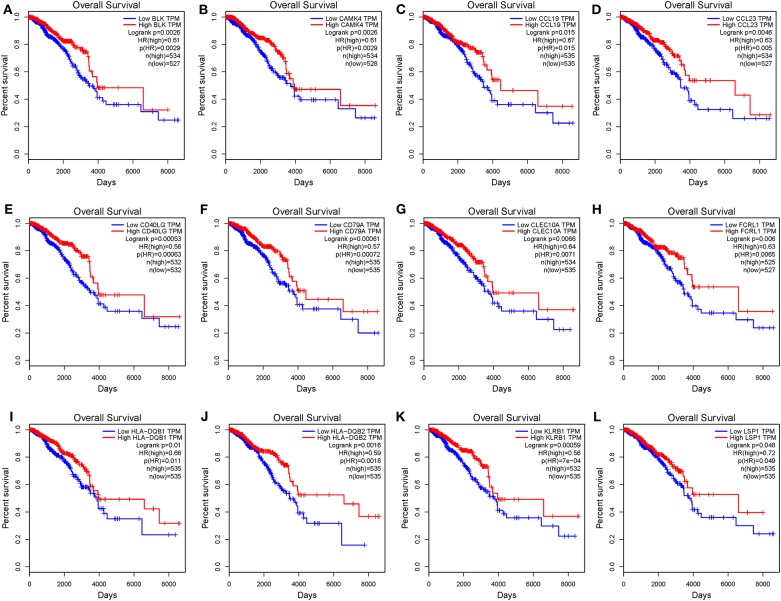
Common DEGs that significantly impact overall survival. **(A–L)** Twelve commonly upregulated DEGs that were significantly associated with better prognosis. OS, overall survival (in days).

**Figure 4:**
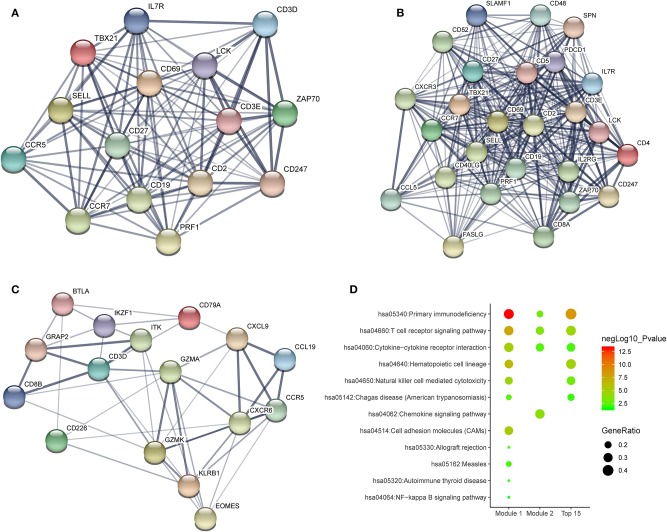
Potential crosstalk between significantly DEGs. **(A–C)** Top 3 modules from a protein-protein interaction network of 129 significantly DEGs. **(D)** The most enriched KEGG pathways in the top 3 modules.

To investigate the potential effects of individual DEGs on OS, we downloaded overall survival curves from the website http://gepia.cancer-pku.cn/. Among all the 403 DEGs, only 129 upregulated genes were significantly associated with OS and predicted better prognosis of patients (Figure 3; *p* < 0.05). What's more, we validated those selected DEGs in the Breast Cancer Metabric data downloaded from cBioPortal (http://download.cbioportal.org/brca_metabric.tar.gz). We drew survival curves using the Graphpad software and found that higher expression of 102 genes was significantly associated with longer overall survival (Figure 6). To explore the crosstalk between these DEGs, we performed protein-protein interactions network analysis using the online tool STRING. By focusing on the genes that had the most connections with other genes, we obtained 3 typical networks, and interestingly, all were associated with immune response. In the first module, CD69, CD27, CD2, CD3E, and LCK were prominent nodes (Figure 4A). For the second module, PDCD1 (PD-1), CD5, SELL, and CD19 had the most connections (Figure 4B). In the last selected module, GZMA, GZMK, CD3D, and KLRB1 were the most important nodes (Figure 4C). Further, we analyzed the related pathways using KEGG and found that the top associated pathways were primary immunodeficiency, T cell receptor signaling pathway and cytokine-cytokine receptor reaction (Figure 4D).

### Aging Modified the Immune Microenvironment as Well as Checkpoints

**Figure 5:**
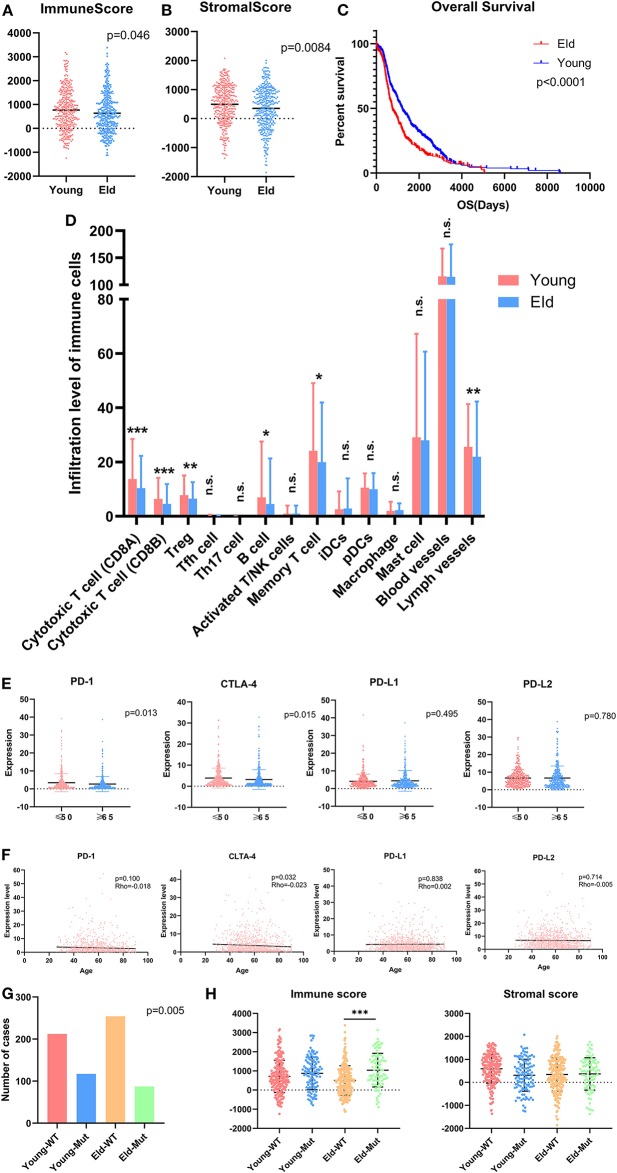
Aging alters the immune microenvironment as well as checkpoint expression levels. **(A,B)** Distribution of immune scores or stromal scores according to the age of breast cancer patients: young group (≤50 years old) vs. eld group (≥65 years old). **(C)** Aging was significantly associated with worse prognosis (*p* < 0.0001). **(D)** Age altered the infiltration of immune cells in the microenvironment, especially T cells, B cells and Treg cells. **(E)** Common checkpoints in breast cancer. **(F)** Association between age and checkpoints in breast cancer. **(G)** Distribution of immune scores in the young or eld group with or without TP53 mutation. *p*-value for chi-square test. **(H)** Distribution of stromal scores in the young or eld group with or without TP53 mutation. WT, wild-type TP53; Mut, TP53 mutation. *p < 0.05, **p < 0.01, and ***p < 0.001 for analysis of variance (ANOVA).

As a strong aging-associated carcinoma (16), we were curious whether aging was associated with the immune/stromal scores of breast cancer patients. We grouped patients older than 64 years old in an eld group and patients younger than 51 years old in a young group. Patients with ages ranging from 51 to 64 years old were excluded from this analysis. We compared the immune/stromal scores in the eld/young groups and found that the median immune score of the young group was higher than that of the eld group (Figure 5A; 768.7 vs. 639.1, *p* = 0.046). In addition, the median stromal score of the young group was also higher than that of the eld group (Figure 5B; 492.3 vs. 350.8, *p* = 0.0084). We also compared immune scores and stromal scores in young and elderly groups in different breast cancer subtypes (Figures 8A,B). Unfortunately, there were no significant differences in two groups among different subtypes, except for the stromal score of the eld group was lower than that of the young group in Luminal A subtype (318.7 vs 592.9, *p* = 0.000947). Kaplan-Meier survival analysis demonstrated that the median overall survival of patients in the young group was longer than that of patients in the eld group (Figure 5C; 1206d vs. 747d, *p* < 0.0001). Our results showed that aging was significantly associated with lower immune/stromal scores and with poor overall survival. To explore the relationship between aging and immune/stromal scores, we compared the infiltration level of immune cells in the tumor microenvironment in the eld/young groups. The results demonstrated that infiltration of cytotoxic T cells, memory T cells, B cells and lymph vessels in the young group was higher than that in the eld group (Figure 5D). However, the infiltration of Treg cells was higher in the young group (Figure 5D). What's more, we added analysis about the infiltration levels of TILs in different breast cancer subtypes. As shown in Figure 7, the infiltration levels of cytotoxic T cells, Treg cells, B cells, memory T cell, iDcs and mast cells in the TME of TNBC patients were higher than that of Luminal A and Luminal B patients, which were consistent with previous study. In addition, we analyzed immune checkpoints, which are highly associated with cancer immunotherapy. The expression levels of PD-1 and CTLA-4 were significantly higher in the young group than in the eld group (Figure 5E; *p* = 0.013, and *p* = 0.015, respectively). We also compared the expression of checkpoints in young and elderly groups in different breast cancer subtypes (Figure 8C). It was disappointing that there were no significant association between age and the expression of checkpoints in different subtypes. We also analyzed the relationship between aging and the expression of PD-1, CTLA-4, PD-L1, and PD-L2 and found that almost all the checkpoints were negatively linearly associated with aging, except PD-L1 (Figure 5F), although only CTLA-4 expression was significantly associated with aging (Figure 5F; *p* = 0.032). Furthermore, TP53 is one of the most common mutations in breast cancer (17), and the number of cases with TP53 mutation in the eld group was lower than that in the young group (Figure 5G; *p* = 0.005). TP53 mutation was also associated with higher immune scores in the eld group (Figure 5H; *p* < 0.001), which may indicate that more mutations were accompanied by a greater immune response due to the production of tumor neoantigens. Moreover, changes in gene mutation frequency might be involved in aging-related changes in the microenvironment.

**Figure 6:**
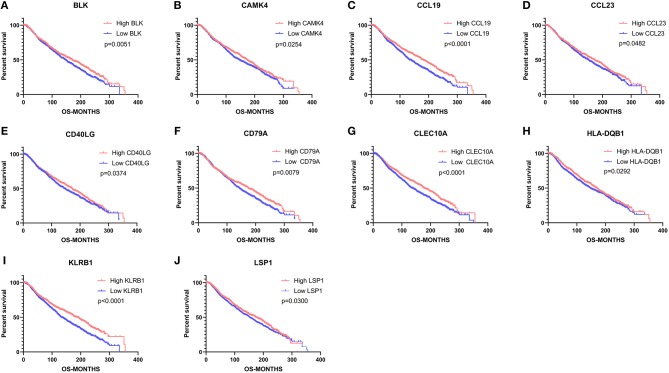
Validation of common DEGs that significantly impact overall survival in the Breast Cancer Metabric data downloaded from cBioPortal. **(A–J)** 10 commonly upregulated DEGs that were significantly associated with better prognosis. OS, overall survival (in months).

**Figure 7:**
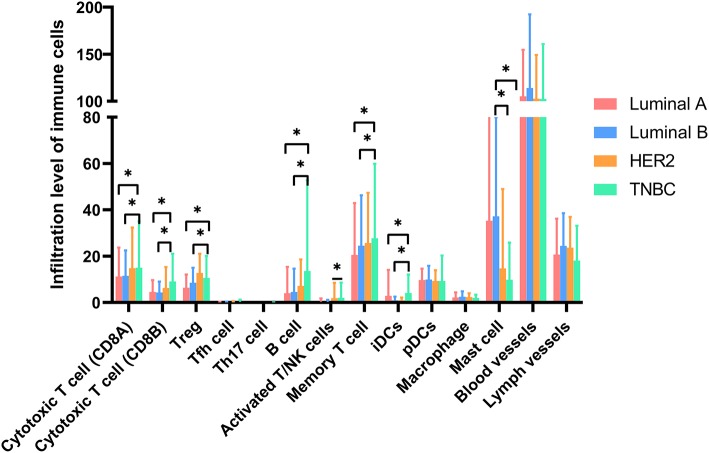
Molecular subtypes altered the infiltration of immune cells in the microenvironment, especially T cells, B cells and Treg cells. *p < 0.05 for multiple *t*-test.

**Figure 8:**
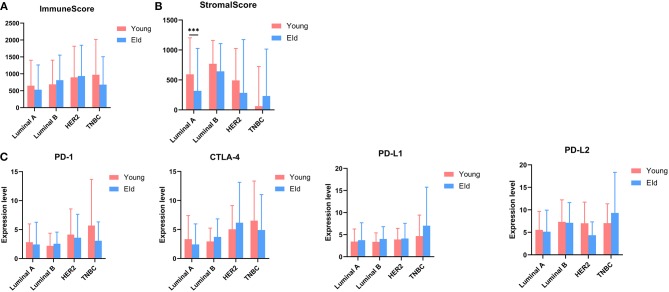
**(A,B)** Distribution of immune scores or stromal scores in different subtypes according to the age of breast cancer patients: young group (≤50 years old) vs. eld group (≥65 years old). **(C)** The expression of common checkpoints in breast cancer subtypes. ***p < 0.001 for multiple *t*-test.

## Discussion

There is growing evidence that abnormal immune/inflammatory responses in the tumor microenvironment are essential mechanisms that promote cancer progression (2). We attempted to excavate correlated genes that were significantly associated with the overall survival of breast cancer patients based on TCGA data. Using the ESTIMATE algorithm, we calculated the immune/stromal scores of each selected patient according to the expression of involved genes in the two groups (9). By comparing gene expression profiles of patients with high or low scores, we identified 403 commonly DEGs (397 genes upregulated, and 6 genes downregulated). We also identified 129 upregulated DEGs that predicted better prognosis of patients, and high immune/stromal scores were found be a protective factor in breast cancer patients. In addition, we verified that aging is related to less infiltration of immune cells and lower expression of immune checkpoints, which contribute to poor prognosis.

In terms of pathological differences, we found that the immune and stromal scores of infiltrating ductal carcinoma were lower than those of infiltrating lobular carcinoma, and metaplastic carcinoma had the highest stromal score, while medullary carcinoma had the highest immune score. Recent studies have demonstrated that different pathological breast cancer subtypes have different genetic characteristics. TP53 and PIK3CA mutations are common in ductal breast carcinoma (18, 19), while mucinous carcinoma lacks PIK3CA mutation (20). ERBB2 mutation is commonly present in both infiltrating lobular carcinoma and mucinous carcinoma (21, 22). TP53 is the most commonly mutated gene in metaplastic breast carcinoma (23), and hypermethylation of the BRCA1 promoter has been detected in medullary carcinoma (24). These differences may result from the heterogeneity of the tumor microenvironment, which has been confirmed by observation of differences in infiltrating lymphocytes in different subtypes of breast cancer (25–27). Tumor-infiltrating lymphocyte (TIL) numbers in infiltrating lobular carcinoma are significantly lower than in infiltrating ductal carcinoma (27), and TILs in medullary carcinoma are primarily CD8+ T lymphocytes (26). Given that immune/stromal scores were positively associated with overall survival of patients, patients with high scores are more likely to have longer disease-free survival and to benefit from immunotherapy. We came to the hypothesis that patients with infiltrating ductal carcinoma or medullary carcinoma might be more sensitive to immunotherapy. However, metaplastic cancer, which is a rare subtype of breast cancer, is poorly responsive to neoadjuvant chemotherapy (28). Patients with metaplastic cancer may benefit from targeted therapy or radiation therapy.

Our results demonstrated that patients with a negative HR status and a positive HER-2 status had the highest immune scores, while the highest stromal scores were observed in patients with both a positive HR status and positive HER2 status. Moreover, TILs were discovered to be a good prognostic value in TNBC but fail in the luminal subtypes (29). This correlation was subsequently independently confirmed in 481 TNBC sample prospectively collected during two phase III adjuvant randomized BC trials (30). Growing evidence focused on immunotherapy for breast cancer has demonstrated that higher numbers of TILs and Treg cells are significantly associated with the TNBC and HER2 overexpression subtypes (31, 32), and more PD-1+ TILs have been found in TNBC and were correlated with higher immune scores (33). In recent clinical trials, immunotherapy, such as blockade of PD-1 (Pembrolizumab), PD-L1 (Durvalumab) and CTLA-4 (Tremelimumab), reached a higher objective response (OR) in TNBC than in HR+ patients (32, 34). The presence of TILs, especially CD8+ T cells, and the expression of checkpoints contributed to the better response to immunotherapy. The infiltration level of TILs could almost be a predictive factor for therapy response (32). Mechanistically, activated HER2 induces the production of CCL2 (C-C Motif Chemokine Ligand 2) through the PI3K-NF-κB axis, promoting recruitment and activation of infiltrated immune cells (35), and TNBC patients were found to have a higher tumor mutation burden (TMB) and to present neoantigens that are correlated with a more effective immunotherapy response (36), while estrogen and estrogen receptor (ER) signaling appears to have little impact on the immune environment (37). With regard to stromal signatures, cancer-associated fibroblasts (CAFs) are one of the most important stromal cell types in the breast cancer microenvironment. CAF-related proteins, such as fibroblast activation protein alpha (FAPα), podoplanin, S100A4, platelet-derived growth factor receptor alpha (PDGFRα), and PDGFRβ, were found to be upregulated in HER2-overexpressing patients (38). The expression of matrix metalloproteinase 2 (MMP2), which facilitates tumor invasion, has been shown to be significantly increased in HER2 subtype patients but not in TNBC patients (39). In ER+ cases, hormone therapy remains the most important therapy; however, acquired resistance to endocrine therapy is a clinical obstacle. Compared with single hormone therapy, a combination of hormone therapy and interleukin-2, interleukin-12 and interferons presented a better anti-tumor effect via synergism with antiestrogens (40). In addition, combining immune checkpoint inhibitors and targeted therapies, such as targeting of PD-1 and PD-L1 combined with HER2-targeted therapy (Trastuzumab), can improve the prognosis of PD-L1-positive patients (41). Above all, immunotherapy is a promising approach for TNBC and HER2-overexpressing subtypes, and patients with the HER2-overexpressing subtype as well as the HR+ and TNBC subtypes can benefit from targeted therapies. Combination therapies with immunotherapy in breast cancer molecular subtypes are worth further investigation.

Among the DEGs identified, we performed overall survival analysis and found that 129 upregulated DEGs were positively associated with better prognosis. However, a previous study on glioblastoma (GBM) obtained the opposite results, reporting that the selected upregulated DEGs were correlated with poor prognosis of patients (42). Similar studies of non–small cell lung cancer (NSCLC) and pancreatic cancer showed that a high immune score predicted a good prognosis (43, 44), which was consistent with our results. The brain is a natural immune exemption zone because of the blood-brain barrier, and thus, its microenvironment is very different from that in breast cancer. Compared with primary breast cancer, paired brain metastases presented significantly lower TIL abundance (45). The expression of the CAF-related proteins stromal podoplanin, stromal PDGFRα and stromal PDGFRβ is reduced in brain metastases (46). However, in GBM, one of the most aggressive brain tumors, the dominant infiltrating immune cells are tumor-associated macrophages (TAMs), which facilitate tumor growth via their proangiogenic and immunosuppressive effects (47). Furthermore, CD8+ TILs are the most common infiltrating immune cells in NSCLC (48). This could partially explain the confusing results in GBM that were not consistent with those in breast cancer. A combination of immunotherapy and chemotherapy (such as atezolizumab plus nab-paclitaxel) was shown to improve the progression-free survival (PFS) and overall survival rate in metastatic TNBC (11, 41), and application of a vaccine against the GBM-specific WT1 peptide significantly increased PFS and overall survival in patients by inducing production of anti-WT1 antibodies and T cell responses (49). In the present study, protein-protein interaction network results showed that upregulated DEGs were primarily associated with immune response, including primary immunodeficiency, T cell receptor signaling pathway and cytokine-cytokine receptor reaction, which predicted a better prognosis via an increased immune response. Together, combination immune and stromal scores or further refinement scores can predict the prognosis of breast cancer or screen out people eligible for immunotherapy.

Aging was significantly associated with lower immune/stromal scores and poor prognosis in breast cancer patients due to reduce infiltration of immune cells and lower expression of checkpoints. The incidence of breast cancer and associated mortality rate increased with age, and almost one half of newly diagnostic breast cancer cases occurred at age 65 and older (50, 51). Barajas-Gómez and colleagues compared the concentration of cytokines between patients and healthy young donors and found that pro-inflammatory cytokine levels, including IL-2, IL-6, and IL-8, were increased in the elderly, whereas anti-inflammatory cytokines, such as IL-4 and IL-10, exhibited decreased expression (52). This low intensity and chronic inflammatory microenvironment promoted tumorigenesis and progression. In elderly patients, proliferation and activity of CD4+ and CD8+ T cells and B cells decreased, while those of immunosuppressive cells increased (53), which formed an immunosuppressive microenvironment that facilitated tumor progression (16). However, few prospective clinical trials have focused on the elderly, and limited studies have demonstrated that older patients benefit from chemotherapy as well as targeted therapies but exhibit more severe cardiotoxicity and bone marrow disorders (54). In addition, anti-PD1 and anti-CTLA-4 immunotherapy was shown to improve OS in both older and younger patients (55). The CheckMate-067 study focused on the combination of nivolumab and ipilimumab for advanced melanoma and showed that patients older than 65 years had a shorter OS than younger patients (56). Above all, the microenvironment changes with age, which may reduce the effects of immunotherapy.

This study still has some shortcomings. The data were only derived from TCGA database and lacked verification with real-world data. We only analyzed transcriptome data, without assessing data associated with non-coding transcriptomes, epigenetic differences or proteomics. We only used bioinformatics analysis, and the study lacked evidence obtained through other methods, such as immunohistochemistry and proteomics, and lacked further validation with a patient-derived xenograft (PDX) mouse model.

In conclusion, based on ESTIMATE algorithm-derived immune/stromal scores, we identified immune microenvironment-related genes that are associated with better patient prognosis. Aging was found to be a risk factor for tumorigenesis and progression and influenced the immune microenvironment and immunotherapy efficacy by altering the number of TILs and checkpoint expression levels. This work may provide new targets for breast cancer treatment that need further investigation in the future.

## Data Availability Statement

Publicly available datasets were analyzed in this study. The data can be found in the TCGA database.

## Author Contributions

BL, RG, and QW designed the study. BL, RG, QW, QY, SZ, and ZX conducted the study, collected and analyzed the data. BL, RG, QW, QY, SiS, SZ, and ShS interpreted the data. BL wrote this article. BL, RG, and QW took responsibility for the integrity of the data analysis. ShS was responsible for editing and submitting this manuscript. All authors approved the submission.

### Conflict of Interest

The authors declare that the research was conducted in the absence of any commercial or financial relationships that could be construed as a potential conflict of interest.

## Abbreviations

BP: biological process
CAFs: cancer-associated fibroblasts
CC: cellular component
CCL2: C-C Motif Chemokine Ligand 2
DAVID, The Database for Annotation: Visualization and Integrated Discovery
DEGs: differentially expressed genes
ER: estrogen receptor
ESTIMATE: Estimation of Stromal and Immune cells in Malignant Tumor tissues using Expression data
FAPα: fibroblast activation protein alpha
GBM: glioblastoma
GO: Gene ontology
HER2: human epidermal growth factor receptor-2
KEGG: Kyoto Encyclopedia of Genes and Genomes
MCODE: the Molecular Complex Detection
MF: molecular function
NSCLC: non–small cell lung cancer
OS: Overall survival
PDGFRα: platelet-derived growth factor receptor alpha
PDX: patient-derived xenograft
PFS: progression-free survival
PPI: protein-protein interaction
STRING: Search Tool for the Retrieval of Interacting Genes
TAMs: tumor-associated macrophages
TCGA: The Cancer Genome Atlas
TIL: tumor-infiltrating lymphocyte
TMB: tumor mutation burden
TME: tumor microenvironment.
